# A dose-escalation ex vivo study on the effects of intracameral benzalkonium chloride in rabbits

**DOI:** 10.1186/s12917-018-1349-8

**Published:** 2018-02-02

**Authors:** Sergi Segarra, Marta Leiva, Daniel Costa, Natàlia Coyo, Maria Sabés-Alsina, José Ríos, Teresa Peña

**Affiliations:** 1 Departament de Medicina i Cirurgia Animals, Facultat de Veterinària, Bellaterra, Spain; 2 Fundació Hospital Clínic Veterinari, UAB, Bellaterra, Spain; 3 Department of Animal Health and Anatomy, Facultat de Veterinària, UAB, Bellaterra, Spain; 4 Medical Statistics Core Facility, IDIBAPS, Hospital Clínic Barcelona, Barcelona, Spain; 5 Biostatistics Unit, Faculty of Medicine, UAB, Bellaterra, Spain

**Keywords:** Endothelial cells, Corneal endothelium, Corneal, Endothelial dystrophy, Endothelial degeneration

## Abstract

**Background:**

Rabbits are currently not a good model for studying diseases of the corneal endothelium because their corneal endothelial cells (CECs) maintain a high proliferative capacity throughout almost all their life. Addressing this particular feature might allow the use of this species for such a purpose. The aim of this study was to evaluate the corneal endothelial injury after intracameral benzalkonium chloride (BAC) injection into rabbit eyes ex vivo, and to establish the most suitable starting dose for an in vivo study aimed at developing an animal model of corneal endothelial disease.

**Results:**

Forty rabbit eyes obtained *postmortem* by transconjunctival enucleation were divided into 8 groups according to the injected compound: Control (no injection), BSS, and increasing BAC concentrations (0.005%, 0.01%, 0.025%, 0.05%, 0.1% and 0.2%). At 0, 6, 24 and 48 h, ophthalmologic examination of the anterior segment, pachymetry and specular microscopy were performed, and corneas were finally vital-stained and observed under the light microscope to assess the CECs morphology and mortality rate. When compared to BSS, CECs density started to decrease significantly at 0.025% BAC concentration, while mean cell area, corneal edema and corneal thickness began to increase significantly at 0.05%, 0.005% and 0.1% BAC concentrations, respectively. Concentrations of 0.05% BAC and above caused significant increases in CECs pleomorphism (decreased hexagonality) and mortality, compared to control and BSS.

**Conclusions:**

Ex vivo intracameral BAC injection induces corneal endothelial toxicity in rabbits. However, confirmatory in vivo studies are required to develop the desired model, with 0.05% BAC being a suggested starting point.

## Background

The corneal endothelium (CE) is a single layer of cells on the inner surface of the cornea, whose main function is controlling the hydration of the cornea, thereby maintaining its transparency. The CE has enough corneal endothelial cells (CECs) to preserve this function throughout life, but a reduction of CECs might occur due to different causes, including endothelial dystrophies, glaucoma, trauma, uveitis, surgeries and stress [1–8]. When there is a decrease in the number of CECs, the remaining cells react increasing their size and altering their morphology, but if cell loss is too great, these compensatory changes are not enough and endothelial decompensation occurs, resulting in corneal edema and partial or complete loss of corneal transparency. Unfortunately, the in vivo proliferative capacity of CECs in humans, primates and small animals is very limited [4–7].

Among the current treatments for diseases affecting the CE in humans, several types of endothelial keratoplasty techniques have been described, such as Descemet stripping (automated) endothelial keratoplasty (DSEK/DSAEK), and Descemet membrane endothelial keratoplasty (DMEK). In addition, the use of cultivated CECs, by either CECs sheet transplantation or CECs injection therapy combined with the application of a Rho-kinase (ROCK) inhibitor, has been also reported [5, 9, 10].

The ideal animal model for the development of techniques to repair endothelial lesions should be anatomically and physiologically similar to the human model. Pigs, dogs and cats are commonly used animal models in ophthalmology research, especially in retinal studies, but they are not as commonly used in corneal research. Several experimental animal models for the development of a tissue-engineered CE have been described, including pig, cat, monkey, rat, mouse and rabbit [3, 10, 11]. Because of its high availability, low cost, small size and easy handling, rabbits are very frequently used in research, including research in human ophthalmology [10, 12, 13]. However, rabbits are currently not a good model for studying diseases of the CE because their CECs maintain a high proliferative capacity throughout almost all of their life [6, 10, 11, 13]. Addressing this particular feature might allow the use of this species for such a purpose.

Benzalkonium chloride (BAC), a quaternary ammonium compound, is the most commonly used preservative in ophthalmic preparations at concentrations ranging between 0.004% and 0.025% [7, 14–19]. Topical high BAC concentrations in rabbits, however, have toxic effects on the cornea affecting the integrity of the CE as well as other ocular structures. Furthermore, topical BAC has been shown to induce tear film drying, thickening of corneal epithelium, loss of goblet cells, inflammatory cell infiltration and proliferation in the conjunctiva, and pro-apoptotic effects in conjunctival or trabecular meshwork cells [14–17, 20–23]. In human beings, cases of corneal toxicity have been reported after intraocular exposure during cataract surgery to a BAC preserved viscoelastic [4, 24] or to a balanced salt solution (BSS) also preserved with BAC [25].

Effects of intracameral BAC injection in vivo and in vitro have been previously reported in rabbits [8, 26], although to the authors’ knowledge, the toxic effects of intracameral BAC injection on the CE in rabbits have not been evaluated ex vivo combining specular microscopy and vital staining. The aim of this study was twofold. Firstly, to evaluate the endothelial injury after a BAC intracameral injection into rabbit eyes ex vivo, and secondly, to establish the most suitable starting dose to obtain a repeatable and reproducible in vivo model of endothelial disease*.*

## Methods

### Animals and sampling

The study included a total of 40 rabbit eyes obtained *post-mortem* by transconjunctival enucleation from 20 healthy adult rabbits (New Zealand White, New Zealand White/California cross, New Zealand White/European rabbit cross) of different gender, weighing 2 to 3 kg, between 8 and 12 weeks of age. Two rabbit slaughterhouses provided the animals (Escorxador Industrial de Conills J. Grau SL, Calaf, Barcelona, Spain; and Mularcun SL, Xert, Castelló, Spain). Eyes were enucleated immediately after sacrifice in the slaughterhouse itself. Thereafter, a pack of sterile gauzes were introduced in a sterile container, the eye was placed on top, and 5 mL of cooled Ringer’s lactate solution was instilled and spread over it, creating a moist-chamber. The container was immediately closed, identified and cold transported to the Ophthalmology laboratory of the Veterinary School of the Universitat Autònoma de Barcelona (UAB).

### Preliminary assessment

The preliminary assessment was performed within 6 h from enucleation, by means of slit-lamp biomicroscopy (SL-15 Portable Slit-Lamp Biomicroscope®, Kowa Co. Ltd., Tokyo, Japan), specular microscopy and corneal pachymetry (Specular Microscope SP-2000P®, Topcon, Tokyo, Japan). Ophthalmic examination of the cornea and anterior segment was performed on all eyes in a dark room. Only eyes with no evidence of corneal and/or anterior segment disease were included in the study (*n* = 40). Eyes were placed in a homemade methacrylate eyeball holder and examined using the non-contact specular microscope in an automatic mode. All procedures were performed by the same investigator maintaining a working distance of 25 mm. Initially, eyes were excluded from the study if they presented corneal decompensation, damaged CECs, CECs loss, or presence of inflammatory cells on the CE. Three microphotographs were obtained from the central area of the cornea. Thirty well-defined CECs from each photomicrograph were analyzed to obtain the mean cell area (MCA; expressed in μm^2^) and the endothelial cell density (ECD; expressed in number of cells/mm^2^). Central corneal thickness (CCT; expressed in μm) was measured using the digital pachymeter of the same specular microscope in automatic mode.

### Intracameral injections

Selected eyes (*n* = 40) were classified into 8 groups (*n* = 5) according to the substance injected: Control group (no injection), comparison or BSS group [injection of BSS (BSS sterile irrigation solution, Laboratoris Alcon, el Masnou, Barcelona, Spain)], and 6 Experimental groups (0.005%, 0.01%, 0.025%, 0.05%, 0.1% and 0.2% BAC). The different BAC compounds used in the study were prepared under sterile conditions (Farmàcia Xalabarder, Barcelona, Spain), with BSS as a diluent. All intracameral injections were performed by the same investigator at the corneal limbus, under magnification loupes (Loupes 4×, Zeiss, Germany) and using a 27 G needle. The needle was removed immediately after the injection. One or two drops of aqueous humor were allowed to passively egress through the hub of the needle, then 0.1 mL of the appropriate compound were carefully injected into the anterior chamber. Pressure was then applied at the injection site with conjunctival forceps for 20 s to avoid fluid leakage.

### Post-injection assessment

Biomicroscopy, pachymetry and specular microscopy were also performed at 6, 24 and 48 h post-injection, following the preliminary assessment protocol. Corneal edema was graded from 0 (absence) to + 4 (very severe) by biomicroscopy. Throughout the experiment, eyes were kept inside the moist chambers under refrigeration at 4 °C.

### Vital staining of corneal endothelium

After the 48 h post-injection assessment, corneas were excised with a 2–3 mm scleral ring, and placed endothelial side up in a Teflon holder to minimize wrinkling and distortion. Damage to CECs was determined by using trypan blue (Trypan blue powder, Sigma-Aldrich, St Louis, MO, USA) and alizarin red S (Alizarin red S powder, Sigma-Aldrich, St Louis, MO, USA) vital stains, following a protocol described elsewhere [27]. A 0.25% trypan blue staining dilution was prepared by diluting 0.025 mg trypan blue powder into 10 mL of 0.9% physiological saline, and a 0.2% alizarin red staining dilution was prepared by diluting 0.02 mg alizarin red powder into 10 mL of 0.9% physiological saline. The pH of the preparations was adjusted to 4.2 in order to achieve optimum dye-laking at the intercellular borders, as described by Taylor et al. [27]. Using this staining technique, damaged cells become permeable to trypan blue and show deep blue staining of their nuclei; and alizarin red allows the visualization of intercellular borders of CECs, as they appear stained in red [27, 28].

After the staining procedure, several radial incisions were made on each sample in order to flatten the cornea before its examination under the light microscope. Corneas were then flat-mounted endothelial side up and evaluated under the light microscope with a 40× objective (Motic® BA210, Motic Group, Spain). For each sample, three photographs of the central cornea at 40× were taken (Nikon Eclipse TE 2000, Nikon Corporation, Tokyo, Japan) and processed with image analysis software (Image J, National Institutes of Health, Bethesda, MD, USA) to measure the mortality rate and the degree of pleomorphism. In each of these images, 30 CECs were evaluated to quantify the number of CECs which had lost their original hexagonal shape, and used to calculate the percentage of hexagonal CECs, or hexagonality. Regarding CECs vitality, if the percentage of nuclei stained in blue –indicating that CECs were devitalized– was 33% or less, mortality was classified as Low; if this percentage was between 34 and 66%, it was considered as Medium level of mortality; and if the percentage of dead CECs was 67% or higher, the image was classified as showing a High mortality level. When appropriate, 10× images were also taken.

### Statistical analysis

No formal calculation of sample size was done due to the characteristics of the experiment (first approach to a new experimental animal model). It was expected that 5 eyes per group would allow obtaining appropriate results data and doing a preliminary exploration of the specific described sections.

Results were described with routine statistical methods and tabulated by group. Categorical and ordinal variables were summarized with absolute frequencies and percentages and mean, standard deviation (SD), and range (minimum, maximum) were used for continuous variables. When applicable, these summaries were provided by time-point of assessment. Percentage of hexagonality and mortality 48 h post-injection were evaluated by one-way ANOVA. Number of cases per group classified as having a Low, Medium or High percentage of dead CECs was analyzed by one-way ANOVA with a non-parametric approach by means rank-transformation. The analysis for longitudinal evaluation was performed using a Generalized Estimating Equations (GEE) approach with an estimation of within-subject correlation from AR(1) approach. These models included group and time as factors with baseline results of independent variable as covariate and the estimation of effect by group was presented by estimated means and their standard error (SE). All statistical analyses were performed with IBM SPSS version 20, Armonk, NY and the significance level alpha was set at 0.05 two-tailed.

## Results

Initially, the Control group (no injection) and the BSS group were compared in order to see if injecting BSS into the anterior chamber could alter the cornea by itself. No significant differences (*p* > 0.05) were found between these groups for any of the parameters studied over time (data not shown), therefore the BSS group was considered to be the comparison group for the study.

The different degrees of corneal edema detected during the study are depicted as means in Table 1. A significant increase in corneal edema was observed in groups 0.005%, 0.01%, 0.025%, 0.05%, 0.1% and 0.2% BAC, when compared to the BSS group (*p* < 0.001).

**Table 1:** Degree of corneal edema after ex vivo intracameral injection of different BAC concentrations into rabbit eyes

	Corneal edema 0 h	Corneal edema 6 h	Corneal edema 24 h	Corneal edema 48 h
BSS	0	0	+ 2	+ 2
0.005% BAC	0	0	+ 1	+ 1
0.01% BAC	0	0	+ 1	+ 1
0.025% BAC	0	+ 1	+ 1	+ 1
0.05% BAC	0	+ 2	+3	+3
0.1% BAC	0	+ 1	+ 3	+ 3
0.2% BAC	0	+3	+ 4	+ 4

[0: no edema; + 1: mild corneal edema; + 2: moderate corneal edema; + 3 severe corneal edema; + 4: very severe corneal edema]

### Specular microscopy

**Fig. 1:**
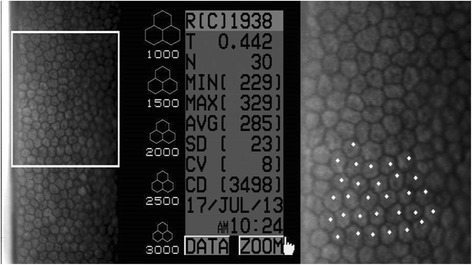
Specular microscopy images with (right) and without (left) zoom including CCT (T), ECD (CD) and MCA (AVG) values of eye 504–2 (Control group – no injection) at time 0 h (SP-2000P specular microscope). Normal rabbit CE was visualized as a monolayer of polygonal cells of uniform size and shape. The zoom image shows 30 well-defined selected CECs marked with white dots, which were used to obtain the MCA and ECD

**Fig. 2:**
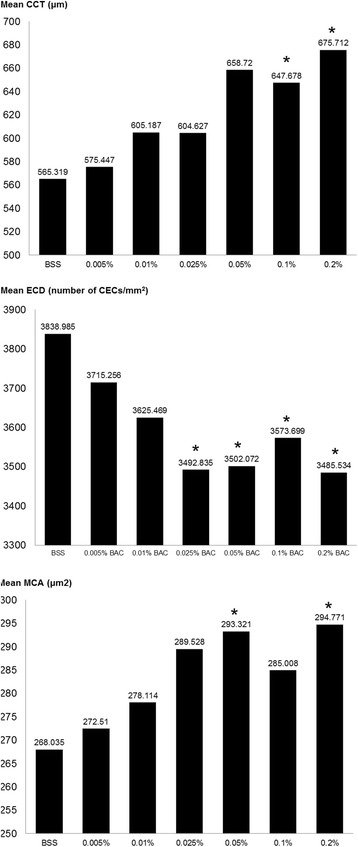
Mean CCT (μm), ECD (number of CECs/mm2) and MCA (μm2) found in rabbit eyes from the different experimental groups. Columns marked with an asterisk (*) represent groups that showed statistically significant differences compared to the BSS group

**Fig. 3:**
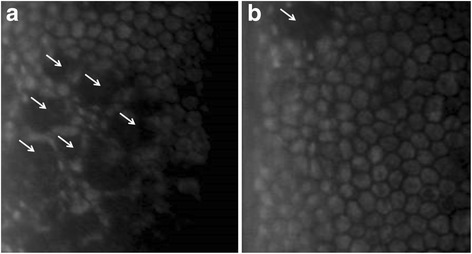
Specular microscopy images of eyes 602–2 (**a**) and 604–2 (**b**), 6 h after injection of 0.025% BAC, showing the presence of CE holes (arrows) (SP-2000P specular microscope)

A total of 695 observations were obtained with the specular microscope (Fig. 1), included in the data set, and statistically evaluated. When compared to the BSS group, a significant decrease in ECD in groups 0.025% (*p* = 0.033), 0.05% (*p* = 0.007), 0.1% (*p* = 0.023) and 0.2% (*p* = 0.004) BAC; and a significant increase in MCA in groups 0.05% (*p* = 0.011) and 0.2% (*p* = 0.005) BAC were seen (Fig. 2). In most of the injected eyes, both with BSS and with increasing concentrations of BAC, specular microscopy evaluation allowed the visualization of holes on the CE (Fig. 3). These changes were observed more frequently with time after injection, regardless of the group.

### Corneal pachymetry and vital-stained microscopic images

**Fig. 4:**
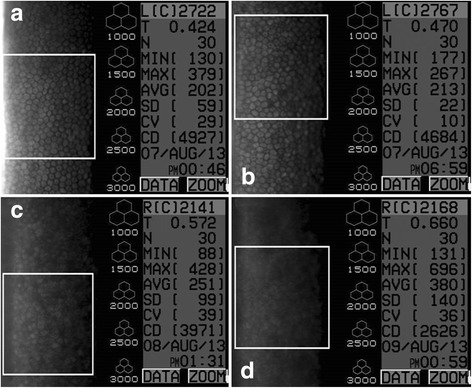
Specular microscopy images including CCT (T), ECD (CD) and MCA (AVG) of eye 803–2 (0.005% BAC) at time 0 (**a**), 6 (**b**), 24 (**c**) and 48 (**d**) hours (SP-2000P specular microscope). During the subsequent assessments, an increase in CCT and MCA, and a decrease in ECD were seen

Although an increase in CCT was observed in all the groups over time, a significant increase in CCT was only seen in groups 0.1% (*p* < 0.001) and 0.2% (*p* = 0.016) BAC when compared to the BSS group (Fig. 2). With time, an increase in CCT and MCA, and a decrease in ECD were observed (Fig. 4).

**Fig. 5:**
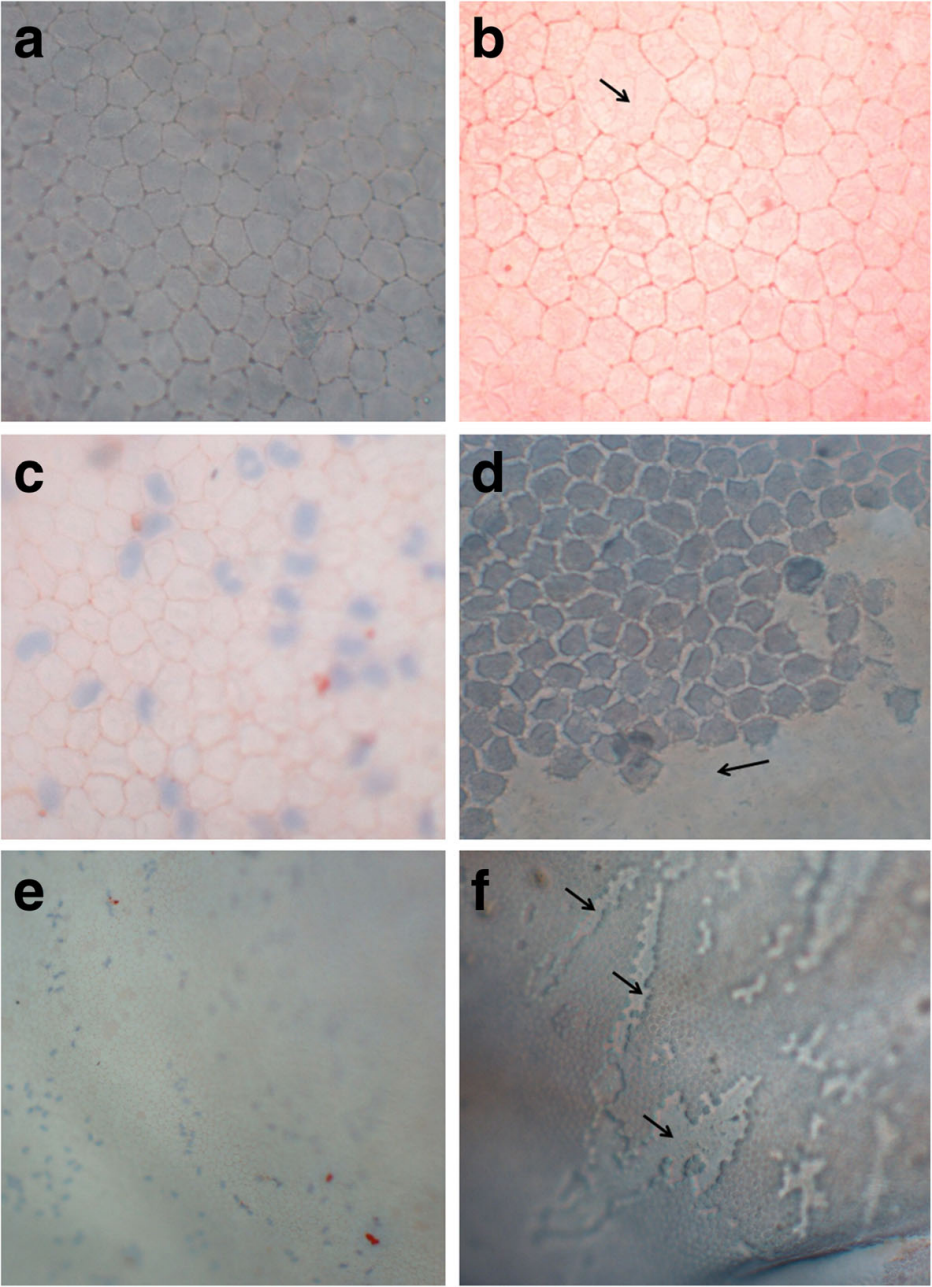
Trypan blue (nuclei of devitalized CECs stained in blue) and alizarin red (staining intercellular borders of CECs in red) vital-stained images of eyes from the study obtained under the light microscope 48 h post-injection. **a**. 40× image of eye 501–2 (Control group – no injection) showing no trypan blue staining; (**b**). 40× image of eye 101–2 (BSS group) showing no trypan blue staining. A giant cell can be observed in the picture (arrow); (**c**). 40× image of eye 805–2 (0.05% BAC) showing trypan blue staining of some CECs and less defined CECs borders; (**d**). 40× image of eye 203 (0.1% BAC) showing trypan blue staining of the totality of CECs and a CE hole (arrow); (**e**). 10× image of eye 603–2 (0.025% BAC): CECs can easily be appreciated and trypan blue staining of some CECs is shown; (**f**). 10× image of eye 203 (0.1% BAC) showing some CE holes (arrows)

The percentages of cell hexagonality and dead CECs seen at 48 h post-injection are summarized in Tables 2 and 3. When compared to Control and BSS groups, significant increases were seen in CECs mortality and pleomorphism (decrease in hexagonal cell population) with concentrations of 0.05% BAC and above. Vital staining also allowed the visualization of holes on the CE (Fig. 5).

**Table 2:** Mean, Standard deviation (SD), minimum (Min) and maximum (Max) values for the percentage of hexagonality in each study group 48 h post-injection

	Mean	SD	Min	Max	*p-*value vs. Control group	*p-*value vs. BSS group
Control group	64.0	5.18	55.0	67.8	–	–
BSS group	66.8	6.04	61.7	75.6	0.758	–
0.005% BAC	43.6	7.47	31.7	52.2	0.031	0.015
0.01% BAC	59.95	11.07	44.2	70.0	0.674	0.478
0.025% BAC	71.0	4.78	65.8	77.2	0.467	0.661
0.05% BAC	**10.7**	**23.84**	**0**	**53.3**	**< 0.001**	**< 0.001**
0.1% BAC	**12.0**	**26.83**	**0**	**60.0**	**< 0.001**	**< 0.001**
0.2% BAC	**0**	**0**	**0**	**0**	**< 0.001**	**< 0.001**

The level of statistical significance (*p*-value) for the values of each group compared to the Control group (no injection) and the BSS group is also shown. Bold text indicates a statistically significant difference with a *p*-value less than 0.01

**Table 3:** Number of cases per group (*n* = 5) classified as having a low (0–33%), medium (34–66%) or high (67–100%) percentage of dead CECs 48 h post-injection

	Low	Medium	High	*p-*value vs. Control group	*p-*value vs. BSS group
Control group	5	0	0	–	–
BSS group	5	0	0	1.000	–
0.005% BAC	4	1	0	0.423	0.423
0.01% BAC	3	1	0	0.346	0.346
0.025% BAC	4	1	0	0.423	0.423
0.05% BAC	**1**	**0**	**4**	**< 0.001**	**< 0.001**
0.1% BAC	**0**	**0**	**3**	**< 0.001**	**< 0.001**
0.2% BAC	**0**	**0**	**5**	**< 0.001**	**< 0.001**

The level of statistical significance (*p*-value) for the values of each group compared to the Control group (no injection) and the BSS group is also shown. Bold text indicates a statistically significant difference with a *p*-value less than 0.01

## Discussion

Endothelial corneal diseases can induce different degrees of visual deprivation that may benefit from corneal surgery. Nowadays, endothelial transplantation is the treatment of choice for this purpose. Endothelial keratoplasty techniques have significantly progressed during the last decade, DSAEK and finally DMEK being their best examples of selectivity [5, 9, 10]. On the other hand, research on drugs directed to improve endothelial health is also increasing [5, 6], and gene therapy could also be applied to corneal endothelial disease in the near future. These research projects could clearly benefit from having a reproducible and repeatable animal model of endothelial disease.

Ideally, an animal model should resemble the human disease in terms of similar response to treatment, similar causation or similar pathophysiological phenotype [29]. In cases where there is more than one alternative, researchers should select the least phylogenetically developed animal available. Despite the persistent in vivo CECs proliferative capacities of rabbit CE, rabbits are frequently used as an animal model for corneal surgery, mainly due to their size, easy handling, short vital cycles and low cost (purchase, housing and maintenance). Rabbits bridge the gap between the small rodent animal models (such as the rat and the mouse) and the larger animal models, often required for pre-clinical, translational research. In the past, partial endothelial dysfunction models have already been described using rabbits and monkeys. Minkowski et al. developed an in vivo model of corneal endothelial dysfunction induced by trans-corneal cryo-injury [3]. This lesion did not selectively affect the CE, but it involved the entire cornea instead. Moreover, within a few days, the CE recovered from the injury. Other models involving CECs scraping have also been described in rabbits and monkeys [5, 6, 30], but surgical intraocular intervention was needed. A CE dysfunction model induced by intracameral injection would consequently represent an easier, cheaper and more repeatable procedure to perform. The results of the present study lead to the speculation that rabbits treated with intracameral BAC could become a suitable model for controlled pre-clinical studies aimed at evaluating the efficacy of novel therapies for managing diseases of the CE.

Several in vivo and in vitro procedures have been described to assess the function and structure of the CE, such as pachymetry, specular microscopy, endothelial vital staining, biomicroscopy, histopathology, and scanning and transmission electron microscopy [8, 10, 23, 27, 31]. Pachymetric measurements reflect the ability of the CE to maintain corneal thickness and dehydration, whereas specular microscopy allows characterization of the CE. The TOPCON SP-2000P® specular microscope provides pachymetric measurements and specular microscopy simultaneously, and gives repeatable and reproducible values for ECD and MCA [32, 33]. In the present study, this device provided clear images of the CE in healthy and mildly abnormal corneas, but obtaining adequate images from moderately to severely edematous corneas was not possible, as it also occurred previously in human studies [33]. In those eyes, vital staining was decisive for the final CE evaluation. As previously described [27, 28], the combined staining of devitalized cells with trypan blue, together with delineation of healthy cells with alizarin red, allowed a good visualization of both damaged and undamaged CECs. In our study we confirmed that, as reported by Taylor et al. [27], adjusting the pH of the alizarin red preparation to 4.2 was of critical importance in order to ensure its effectiveness.

Due to its good safety and efficacy profile, BAC is the most commonly used preservative in ophthalmic preparations. However, long-term use of topical drugs with BAC may damage the cornea [14, 16, 19]. The toxic effect of topical BAC on the ocular surface has been reported by different authors, mainly affecting the corneal epithelium and stroma [14, 15, 17, 18]. Although there are some reports on the toxic effect of topical BAC on the CE in rats [18] and rabbits [14, 23], the studies in rabbits so far have shown some conflicting results. Chen et al. reported an increased CCT, without affecting ECD, after topical application of 0.1% BAC twice daily for four days in an in vivo model [14], whereas Green et al. found no damage to the CE after topical application of 0.133% BAC on deepithelialized corneas [23]. The results of both studies indicate that topical BAC rarely affects the CE unless administered at high and repeated doses and, in such cases, other superficial corneal and/or conjunctival toxic alterations may also appear [14, 15, 17, 18]. As reported previously [26] and confirmed in the herein report, the threshold for CECs BAC-induced intraocular toxicity is lower than when applied topically, thus allowing a more selective damage to the CE using a lower BAC concentration. Therefore, the intracameral administration could be considered the route of choice for inducing a rabbit model of corneal endothelial disease through selective destruction of CECs. This would be a relatively easy technique to obtain a model for studying diseases of the CE in a species like the rabbit which, as stated previously, is very convenient in many aspects for performing pre-clinical studies.

The results of the present study establish a direct relationship between intracameral BAC concentrations and CCT, degree of corneal edema, endothelial mortality and endothelial pleomorphism; and an inverse relationship between intracameral BAC concentrations and ECD and CECs hexagonality. These relationships can be explained by the increase in CCT produced by the reduction in ECD induced, in turn, by the toxic effect of intracameral BAC. The dose dependent increase in MCA can also be explained by the toxicity of intraocular BAC.

Although there are some previous studies on the effects of intracameral BAC on CCT, corneal edema, ECD and MCA [8, 26, 34], there are no reports on the effects on mortality rate, pleomorphism and the presence of endothelial holes. The increase in CCT and corneal edema, found in response to increasing intraocular concentrations of BAC in the present study, is in line with the results of two previously reported studies in rabbits [8, 26]. Although a higher mean CCT was achieved with 0.05% BAC, compared to 0.1% BAC, the 0.05% group did not reach the level of statistical significance probably due to a high standard deviation within the measurements. Moreover, increases in CCT and MCA, together with a decreased ECD, have also been reported in human patients with corneal toxicity secondary to BAC preserved viscoelastic material or BSS used during cataract surgery [24, 25, 34]. The mortality rate increased significantly with higher BAC concentrations, which may have induced a secondary increase in MCA and reduction in hexagonality. Endothelial holes were observed by both specular microscopy and vital staining, which rules out a manipulation artefact during the staining procedure as a possible explanation, and yields the BAC toxic effect as the most probable cause. It is important to mention that in this study the procedure of intracameral injection did not alter the CE, as confirmed by the lack of significant differences between the Control and BSS groups for any of the studied parameters.

Britton et al. defined the threshold for intraocular irritation induced by intracameral BAC injection as 0.03%, based on alterations that occurred in a dose-response manner (conjunctivitis, flare, iritis, and corneal changes) [26]. Despite drawing this conclusion only from variations observed in ophthalmic examination and pachymetric measurements, this threshold is similar to 0.05% BAC, which was the minimum concentration needed in the present study to cause significant endothelial damage. Our statement, however, is also supported by objective measurements obtained through evaluations using specular microscopy and vital staining, which further characterize mortality rate and morphologic changes induced by BAC toxicity.

The results of the present ex vivo study yield that the most suitable starting dose to develop a repeatable and reproducible in vivo model of endothelial disease in rabbits is 0.1 mL of 0.05% BAC. The toxic action of BAC would counteract the proliferative capacity of rabbit CECs, hence making this species more suitable for such a model. Higher BAC concentrations were associated with a rapid and intense corneal edema and a complete destruction of CECs in the present ex vivo study; which in an in vivo study could be associated to severe intraocular inflammation. Nevertheless, direct extrapolations to live animals or to other species should not be made.

## Conclusions

The present study reports the endothelial injury induced after intracameral injection of different BAC concentrations into rabbit eyes ex vivo; thus, encouraging its use for the development of an in vivo animal model of endothelial disease, and establishing 0.05% BAC as the most suitable starting dose for this purpose. These findings also provide additional information about the toxic effect of BAC on the cornea.

## Abbreviations

BAC: Benzalkonium chloride
BSS: balanced salt solution
CCT: Central corneal thickness
CE: corneal endothelium
CECs: corneal endothelial cells
DMEK: Descemet membrane endothelial keratoplasty
DSAEK: Descemet stripping automated endothelial keratoplasty
DSEK: Descemet stripping endothelial keratoplasty
ECD: endothelial cell density
GEE: Generalized Estimating Equations
MCA: mean cell area
ROCK: Rho-kinase
SD: standard deviation
SE: standard error
UAB: Universitat Autònoma de Barcelona

## References

[1] Joyce NC (2005). Cell cycle status in human corneal endothelium. Exp. Eye Res. Elsevier.

[2] Collin SP, Collin HB (1998). A comparative study of the corneal endothelium in vertebrates. Clin Exp Optom.

[3] Minkowski JS, Bartels SP, Delori FC, Lee SR, Kenyon KR, Neufeld AH (1984). Corneal endothelial function and structure following cryo-injury in the rabbit. Invest Ophthalmol Vis Sci.

[4] Bourne WM (2003). Biology of the corneal endothelium in health and disease. Eye.

[5] Koizumi N, Okumura N, Kinoshita S (2012). Development of new therapeutic modalities for corneal endothelial disease focused on the proliferation of corneal endothelial cells using animal models. Exp Eye Res Elsevier.

[6] Koizumi N, Sakamoto Y, Okumura N, Okahara N, Tsuchiya H, Torii R (2007). Cultivated corneal endothelial cell sheet transplantation in a primate model. Invest Ophthalmol Vis Sci.

[7] Rodrigues GN, Laus JL, Santos JM, Rigueiro MP, Smith RL (2006). Corneal endothelial cell morphology of normal dogs in different ages. Vet Ophthalmol.

[8] Means TL, Holley GP, Mehta KR, Edelhauser HF (1994). Corneal edema from an intraocular irrigating solution containing Benzalkonium chloride. Cutan Ocul Toxicol.

[9] Dirisamer M, Parker J, Naveiras M, Liarakos VS, Ham L, van Dijk K (2012). Identifying causes for poor visual outcome after DSEK/DSAEK following secondary DMEK in the same eye. Acta Ophthalmol.

[10] Proulx S, Brunette I (2012). Methods being developed for preparation, delivery and transplantation of a tissue-engineered corneal endothelium. Exp Eye Res.

[11] Van Horn DL, Sendele DD, Seideman S, Buco PJ (1977). Regenerative capacity of the corneal endothelium in rabbit and cat. Invest Ophthalmol Vis Sci.

[12] Werner L, Chew J, Mamalis N (2006). Experimental evaluation of ophthalmic devices and solutions using rabbit models. Vet Ophthalmol.

[13] Song J-S, Heo J-H, Kim H-M (2012). Protective effects of dispersive Viscoelastics on corneal endothelial damage in a toxic anterior segment syndrome animal model. Invest Ophthalmol Vis Sci.

[14] Chen W, Li Z, Hu J, Zhang Z, Chen L, Chen Y (2011). Corneal alternations induced by topical application of benzalkonium chloride in rabbit. PLoS One.

[15] Labbé A, Pauly A, Liang H, Brignole-Baudouin F, Martin C, Warnet J-M (2006). Comparison of toxicological profiles of benzalkonium chloride and polyquaternium-1: an experimental study. J Ocul Pharmacol Ther.

[16] Bagnis A, Papadia M, Scotto R, Traverso CE (2011). Antiglaucoma drugs: the role of preservative-free formulations. Saudi J Ophthalmol.

[17] Liang H, Baudouin C, Pauly A, Brignole-Baudouin F (2008). Conjunctival and corneal reactions in rabbits following short-and repeated exposure to preservative-free tafluprost, commercially available latanoprost and 0.02% benzalkonium chloride. Br J Ophthalmol.

[18] Pauly A, Brignole-Baudouin F, Labbé A, Liang H, Warnet J-M, Baudouin C (2007). New tools for the evaluation of toxic ocular surface changes in the rat. Invest Ophthalmol Vis Sci.

[19] Xiong C, Chen D, Liu J, Liu B, Li N, Zhou Y (2008). A rabbit dry eye model induced by topical medication of a preservative benzalkonium chloride. Invest Ophthalmol Vis Sci.

[20] Baudouin C, Labbé A, Liang H, Pauly A, Brignole-Baudouin F (2010). Preservatives in eyedrops: the good, the bad and the ugly. Prog Retin Eye Res.

[21] Liang H, Brignole-Baudouin F, Rabinovich-Guilatt L, Mao Z, Riancho L, Faure MO (2008). Reduction of quaternary ammonium-induced ocular surface toxicity by emulsions: an in vivo study in rabbits. Mol Vis.

[22] Ichijima H, Petroll WM, Jester JV, Cavanagh HD (1992). Confocal microscopic studies of living rabbit cornea treated with benzalkonium chloride. Cornea.

[23] Green K, Hull DS, Vaughn ED, Malizia AA, Bowman K (1977). Rabbit endothelial response to ophthalmic preservatives. Arch Ophthalmol.

[24] Hughes EH, Pretorius M, Eleftheriadis H, Liu CSC (2007). Long-term recovery of the human corneal endothelium after toxic injury by benzalkonium chloride. Br J Ophthalmol.

[25] Liu H, Routley I, Teichmann KD (2001). Toxic endothelial cell destruction from intraocular benzalkonium chloride. J Cataract Refract Surg.

[26] Britton B, Hervey R, Kasten K, Gregg S, McDonald T (1976). Intraocular Irritation evaluation of benzalkonium chloride in rabbits. Ophthalmic Surg.

[27] Taylor MJ, Hunt CJ (1981). Dual staining of corneal endothelium with trypan blue and alizarin red S: importance of pH for the dye-lake reaction. Br J Ophthalmol.

[28] Park S, Fong AG, Cho H, Zhang C, Gritz DC, Mian G (2012). Protocol for vital dye staining of corneal endothelial cells. Cornea.

[29] Rang HP, Dale MM, Ritter JM, Flower RJ, Henderson G. Method and measurement in pharmacology. Rang Dale’s Pharmacol. Elsevier; 2016. p. 91–100.

[30] Okumura N, Koizumi N, Ueno M, Sakamoto Y, Takahashi H, Tsuchiya H (2012). ROCK inhibitor converts corneal endothelial cells into a phenotype capable of regenerating in vivo endothelial tissue. Am J Pathol.

[31] Hull DS (1979). Effects of epinephrine, benzalkonium chloride, and intraocular miotics on corneal endothelium. South Med J.

[32] Bovelle R, Kaufman SC, Thompson HW, Hamano H (1999). Corneal thickness measurements with the Topcon SP-2000P specular microscope and an ultrasound pachymeter. Arch Ophthalmol.

[33] Cheung SW, Cho P (2000). Endothelial cells analysis with the TOPCON specular microscope SP-2000P and IMAGEnet system. Curr Eye Res.

[34] Eleftheriadis H, Cheong M, Sandeman S, Syam PP, Brittain P, Klintworth GK (2002). Corneal toxicity secondary to inadvertent use of benzalkonium chloride preserved viscoelastic material in cataract surgery. Br J Ophthalmol.

